# Three Types of Demyelination, Perivenous, Confluent, and Perineuronal Nets-Rich in a COVID-19 Patient With Meningoencephalomyelitis

**DOI:** 10.7759/cureus.51049

**Published:** 2023-12-24

**Authors:** Rei Asano, Koji Hayashi, Ei Kawahara, Mamiko Sato, Toyoaki Miura

**Affiliations:** 1 Department of Rehabilitation, Fukui General Hospital, Fukui, JPN; 2 Department of Pathology, Fukui General Hospital, Fukui, JPN

**Keywords:** myelitis, meningitis, encephalitis, medulla oblongata, cytotoxic t cell, venule, perineural nets, confluent demyelination, perivenous demyelination, covid-19

## Abstract

Neurologic symptoms are common in COVID-19, and a variety of neuropathological changes have been reported. One of the important neuropathological findings is demyelination. However, the underlying pathogenesis of demyelination remained poorly understood. We witnessed a case of COVID-19 with distinct types of demyelination in the cerebrum, medulla oblongata, and spinal canal, who died of sepsis. The postmortem examination showed the solitary massive demyelination in the medulla oblongata. The massive lesion was filled with components of perineuronal nets. In the spinal canal, confluent demyelination in bilateral lateral and dorsal funiculi was detected over the entire length from C1 to S5, which was maximum at the level of cervical spinal canal stenosis. Demyelination in the cerebrum was mainly perivenular, and augmented in the area of lacunar infarcts and dilated perivascular spaces. Considering the distribution patterns of the following three types of demyelination, the traces of viral spreading could be highlighted. Discontinuous perivenous demyelination in the cerebrum showed the result of hematogenous spreading. Longitudinal confluent demyelination of the spinal cord should be the picturesque of the trace of axonal spreading. The distribution of demyelination was possibly modified by the underlying diseases, diabetes mellitus, hypertension, and spinal canal stenosis.

## Introduction

Neurologic symptoms are common in coronavirus disease 2019 (COVID-19). Magnetic resonance images (MRI) showed encephalomyelitis including encephalitis, acute disseminated encephalomyelitis (ADEM), and myelitis in dorsal and lateral columns [1,2]. Thus, the neuropathological features of COVID-19 patients seem to be too diverse to understand the underlying pathogenesis and have been controversial. However, postmortem pathological studies seem to share a couple of histological changes. Several specific changes including ADEM-like pathology, microvascular injury, and microvascular change with cytotoxic lymphocytes (CTLs) infiltration, were reported [3,4]. Furthermore, immunohistochemistry showed CD8+CTLs in perivascular space and the parenchyma, and luxol fast blue showed demyelination [3]. The key features of microvascular injury, demyelination, and CTL-mediated immune reaction could provide us better understanding of COVID-19-related encephalomyelitis.

The virus selectively infects cells based on the expression of the corresponding cell receptor, angiotensin-converting enzyme 2 (ACE2) [5]. In the central nervous system, endothelial cells, pericytes, vascular smooth muscle cells, neuronal cells, astrocytes, and oligodendrocytes express ACE2, and the high expression in inflammation, hypoxia, or other preexisting diseases raises the risk of infection by modifying the cell tropism of the virus [5]. One assumes that microvascular injury is caused by the high level of free angiotensin II, which shares ACE2 with the virus, around the infected endothelial cells by the effect of nonspecific inflammation [6]. Pericytes of capillaries and post-capillary venules [6], which express ACE2 highly, also participate in vascular injury [6].

In addition to the hypothesized nonspecific reaction, specific immune reactions by CTLs should be participated. CTL-based immune reactions are well known to be initiated at the recognition of the virus antigen presented on class I MHC antigen (MHC I) on the cell surface. Therefore, the real targets of the viral injury should be the cells, which express both ACE2 and MHC I. Neuronal cells do not express MHC I normally and evade immunosurveillance. However, in inflammatory situations, a low number of neuronal cells express MHC I. MHCI loads the processed viral antigen, followed by an immune reaction of CTLs. Injured cells release the virus particles to the environment. The virus infects the adjacent astrocytes and oligodendrocytes, which highly express ACE2. Astrocytes and oligodendrocytes highly express MHC I in the inflammatory environment [7]. Damages to astrocytes in COVID-19 were also reported [8], which decreased in supportive function of vascular and neuronal integrity [3], in addition to damage to oligodendrocytes, which causes demyelination.

Demyelination is defined as the marked damage of myelin despite a relatively preserved axon [9]. The major CNS demyelinating diseases are multiple sclerosis (MS) and ADEM. Then, pathology-based diagnosis has been regarded as the final diagnosis. ADEM is pathologically characterized by the sleeves of perivenous demyelination and MS shows confluent demyelination [8]. The distinct two types of demyelination are used as the hallmark of the two different diseases and the difference suggests the distinct pathogenesis [8]. Although the two distinct types were not clearly described in the reported cases of COVID-19, different types of demyelination including perivenular demyelination, suggested that the mechanisms of COVID-19-related pathology of CNS are clearly multifactorial [3].

We describe here details of a case of COVID-19-related meningoencephalomyelitis with diffuse venular inflammation cuffed with CTLs, and demyelination of the cerebrum, medulla oblongata, and spinal cord. And clarified the possible different pathogenesis. Three types of demyelination, perivenous-, confluent- and novel types of perineural nets rich-demyelination. We will further discuss the cell tropism of SARS-CoV-2 and CTL based on the expression of ACE2 and MHC I compared with the present distribution patterns of the pathological changes.

## Case presentation

**Figure 1:**
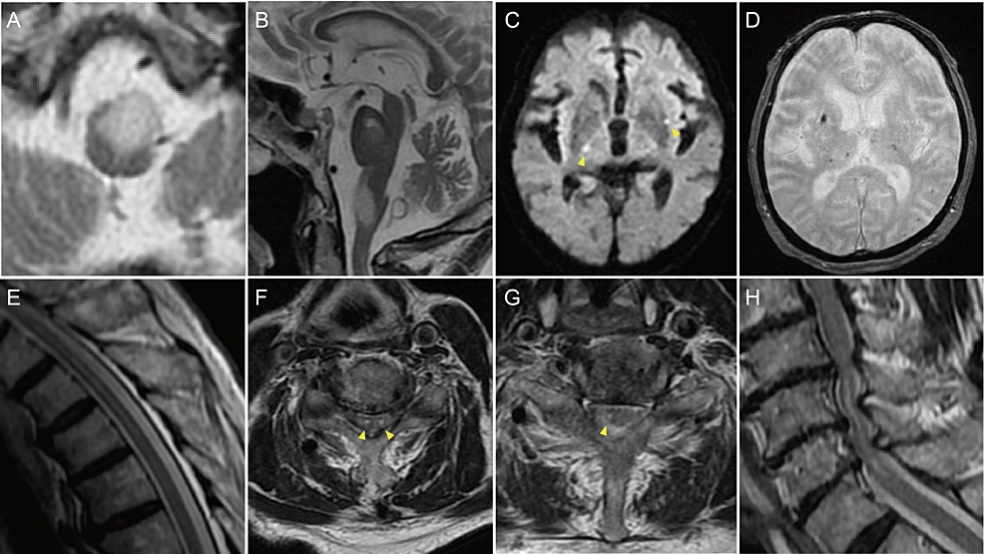
MRI. (A, B) Axial and sagittal T2 images showing massive lesion in the medulla oblongata. (C) Axial diffusion images showing multiple hyperintensity lesions (arrowheads). (D) Axial T2* images showing multiple low-density lesions. (E) Sagittal T2 images showing longitudinal hyperintensity of lateral funiculus from C2 to S5. (F, G) Axial T2 images showing hyperintensity of bilateral lateral funiculi from C6 to T1 and of right lateral funiculi from C2 to C6 and T1 to T7 (arrowheads). (H) Sagittal T2 images showing spinal canal stenosis and hyperintensity area at the level of C5/6 and C6/7.

A male in his early 80s was brought by an ambulance complaining of loss of consciousness with a high fever. Chest CT showed bilateral pneumonia. Physical examination and a laboratory test showed that he had hypertension and diabetes mellitus. Since a test for SARS-CoV-2 by gene amplification was positive, he was transferred to the hospital designed for conventional infectious diseases. He was treated by remdesivir, dexamethasone, and antibacterial medications for 11 days. Although the pneumonia has been improved, cognitive impairment and dysphagia, which has not been noticed before COVID-19 pneumonia, emerged. He was retransferred to the Department of Rehabilitation of the original hospital. Paralysis and spasticity in the right lower limb were also pointed out. The brain CT taken on day 22 showed old infarction in the pons, thalamus, and basal ganglia. MRI taken on Day 28 showed a massive lesion with a high T2 star signal in the medulla oblongata (Figure 1A), which was suspected to be a low-grade glioma or a demyelinating lesion including ADEM after virus infection. In addition to old infarction, multiple lesions of punctate bleeding and infarcts were shown (Figures 1C, 1D). The neurologic manifestations did not seem to be so severe for the big mass in the brain stem. He was retransferred to the Department of Neurology in a university hospital to workup on Day 32. MRI did not show a mass in the spinal cord but did longitudinal T2 hyperintensity of lateral funiculus from C2 to S5 (Figure 1E), and in the transverse section, patchy T2 change was in bilateral lateral funiculi especially denser signals in the left lateral funiculus (Figures 1F, 1G). In addition, severe spinal canal stenosis was pointed out at the level of C5/6 and C6/7 (Figure 1H) as a possible cause of hyperreflexed tendons in the limbs and foot clonus. One month later he came back to the original hospital. MRI on Day 67 showed the mass in the medulla oblongata diminished its size slightly (Figure 1G), and the size remained unchanged on Day 80. Multiple lesions of lacunar infarction with or without bleedings appeared at different places in the brain every time the MRI was taken (Figure 1H).

Blood test results showed elevated C-reactive protein (1.53 mg/dL), IgG (1934 mg/dL), IgM (244 mg/dL), and sIL2-R (838 U/mL) and anti-aquaporin 4 and anti-myelin oligodendrocyte glycoprotein antibodies were negative. Cerebrospinal fluid (CSF) analysis showed elevated protein (135.8 mg/dL) and normal results for cell count (3/µL), myelin basic protein, oligoclonal bands, and IgG index. A tumor cell was not detected by a cytopathological test in CSF.

He underwent mPSL pulse therapies (two cycles for 1,000 mg/day, three days) but the symptoms were not improved. Their general condition was gradually worsened by urinary tract infection and aspiration pneumonia. He died of septic shock on Day 91.

Autopsy findings

An autopsy was conducted at the Department of Pathology, Fukui General Hospital, and the following brain cuttings were at the Department of Pathology, University of Fukui. The brain weighed 1,110 g.

Medulla oblongata

**Figure 2:**
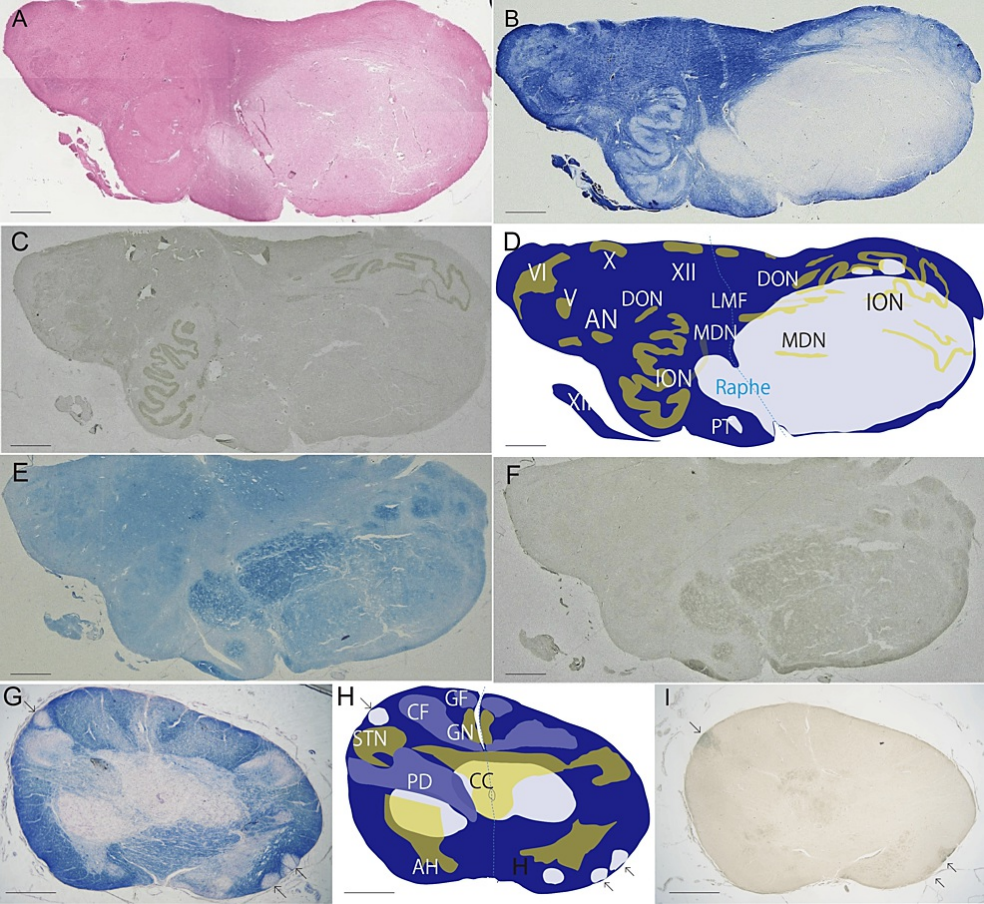
Massive demyelination in medulla oblongata. (A) The lesion is stained pale with hematoxylin and eosin (H&E) stain. (B) The lesion shows marked myelin pallor with Klüver-Barrera (KB) stain. (C) Left inferior olive nucleus and a part of left medial accessory nucleus involved in the lesion are faintly seen with synaptophysin immunoperoxidase. (D) Schematic presentation of myelin pallor and nuclei (ochre and yellow) based on combination of KB (B) and synaptophysin (C). XII, nucleus of hypoglossal nerve; X, dorsal nucleus of vagal nerve; V, spinal tract nucleus of trigeminal nerve; AN, ambiguous nucleus, ION, inferior olivary nucleus; DON, dorsal accessory olivary nucleus; MDN, medial accessory olivary nucleus; PT, pyramidal tract. (E) The lesion is rich in acid mucopolysaccharide. Alcian blue stain. (F) The lesion is also positive for iron diamine, which stains sulfated proteoglycan. The boundary between medulla oblongata and spinal cord at the level of pyramidal decussation. Right pyramidal tract at the level of pyramidal decussation and bilateral cuneate and gracile fasciculi show mild myelin pallor. Low end of massive lesion of myelin pallor with peripheral processes (arrows) (G). KB stain. (H, I) Schematic presentation of Figure 2G. CF, cuneate fasciculus; GF, gracile fasciculus CN, cuneate nucleus; GN, gracile nucleus; STN, spinal trigeminal nucleus; CC, central canal. PD, pyramidal decussation; AH, anterior horn of spinal canal. Arrows indicate processes of the massive lesion of myelin pallor. Scale bar = 2 mm.

There was a well-demarcated lesion in the medulla oblongata. Hematoxylin and eosin (H&E) stain of the section showed that the lesion is oligocellular and homogeneously faintly stained with eosin (Figure 2A) and Klüver-Barrera (KB) stain showed myelin pallor (Figure 2C). The lesion mainly occupied the left ventral half of the medulla oblongata (Figures 2A-2G). Although KB stain could not enable us to know if the lesion affected the inferior olivary nucleus or pushed it back to the dorsal side, the immunoperoxidase for synaptophysin showed a half of the left olivary nucleus was in the lesion (Figure 2D). Moreover, macroscopically grey matter could be observed faintly in fragments (Figure 2B). Finally, the lesion is concluded to mainly include the ventral half of the medulla; pyramidal tract, inferior olivary nucleus, nucleus paragigantocellularis, arcuate nucleus, medial lemniscus, vagal and hypoglossal nerves inside of the medulla oblongata (Figure 2E).

The lesion showed slightly expansive growth posteriorly and laterally with distortion of the raphe despite its oligo-cellularity and the decreased glial supporting network. Then, it was presumed that some non-fibrillary extracellular matrix could be there. The result showed that colloidal iron stain, which is a sensitive stain for acid mucopolysaccharide, highlighted the lesion (Figure 2F), and high-iron diamine staining for sulfated proteoglycan stained specifically for the lesion (Figure 2G). Since in the central nervous system, only hyaluronic acid and chondroitin sulfate among acid mucopolysaccharides exist normally as a perineuronal network the matrices in the lesion would be hyaluronic acid and matrix-type chondroitin sulfate-based proteoglycan. Colloidal iron and high-iron diamine stains did not show dense positivity in any other lesions including the spinal cord, pons, and cerebrum.

**Figure 3:**
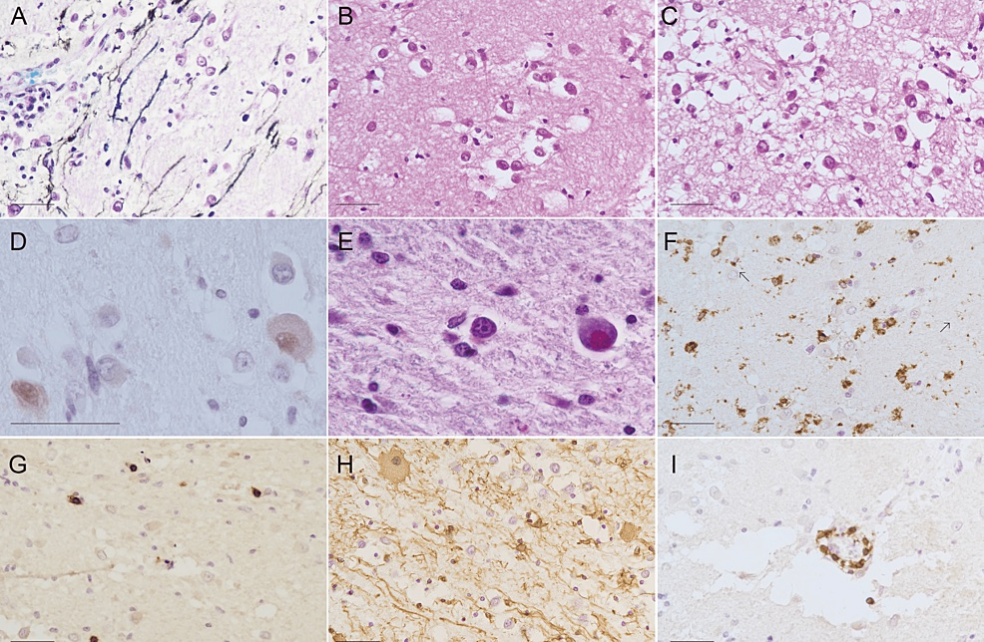
Histopathology of the massive demyelinating lesion in medulla oblongata. (A) Demyelinated axons in the mucinous matrix. Double stain with KB stain and neurofilament peroxidase. (B, C) Representative histopathology with H&E stain. Round cells show vacuolation around cells (B). (D) NeuN-positive neuronal cells in the right inferior olivary nucleus. NeuN immunoperoxidase. (E) Lipofuscin-containing neuronal cells. PAS stain. (F) Sparce network of astrocytic endfeet and gemistocytic reactive astrocytes (arrows). GFAP immunoperoxidase. (G) CD68+ activated microglia. CD68 immunoperoxidase. (H) Cytotoxic T-cell (CTL) infiltration. CD8 immunoperoxidase. (I) Perivenular CTLs. CD8 immunoperoxidase. Scale bar = 40 µm.

Myelin pallor was prominent, and axons are preserved well in the mucinous matrix (Figure 3A), leading to the conclusion that the lesion of myelin pallor was demyelination. The main cellular components which were observed with H&E stain were round cells with large pericellular vacuolation and glial cells with small nuclei in the sparse glial network (Figures 3B, 3C). All the vacuolating round cells were also negative for glial acidic fibrillary protein (GAFP), synaptophysin, chromogranin A, vimentin, Olig2, S100, CD34 and Ki67. NeuN was positive for a few vacuolating cells (Figure 3D). Furthermore, unaffected neuronal cells of the inferior olivary nucleus had numerous lipofuscin granules, and PAS-positive lipofuscin-containing neuronal cells are also found in the periphery of the massive lesion (Figure 3E). A few chromogranin A-positive degenerated neuronal cell was detected. The vacuolating round cells were inferred to be survived neuronal cells, who had scarce neuronal phenotype, in the demyelinated lesion. Glial cells were CD68+ microglia, and CD68 immunoperoxidase revealed their shape was ramified and ameboid (Figure 3F). CD8+ CTLs are sparsely scattered in the parenchyma (Figure 3G). GFAP+ astrocytes decreased in number and the preserved astrocytes was swollen and gemistocytic (Figure 3H). In addition to the massive demyelination perivascular lesions were prominent in the center of the lesion. Perivascular areas were collapsed and showed large clefts around vessels. The vessels, mainly venules which were identified with an ɑ-smooth muscle cell actin-positive pericyte [10] or a few smooth muscle cells and without elastic lamina, were cuffed with CTLs (Figure 3I) and macrophages. A few CD4-positive helper T lymphocytes and CD20+ B cells were found around vessels but not found in the parenchyma. CD34 immunoperoxidase showed marked decrease in capillaries compared to the surrounding white matter and grey matter. Finally, the well demarcated lesion of the medulla oblongata was concluded to be so called demyelinating pseudotumor [11], which has not been defined histologically. Since the histology of the present demyelinating pseudotumor was characterize by richness of perineural nets including chondroitin sulfate and hyaluronic acid, it is tentatively referred to be perineural nets-rich type demyelination.

**Figure 4:**
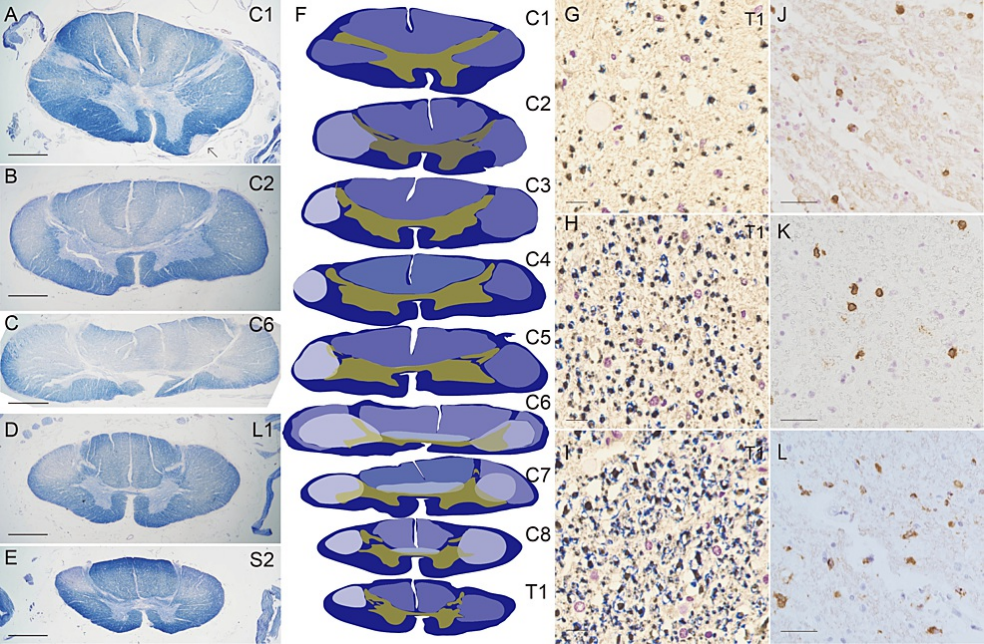
Longitudinal demyelination of spinal cord. KB stain at the level C1 (A), C2 (B), C6 (C), L1 (D), and S2 (E). Right lateral funiculus shows mild to severe myelin pallor consecutively in total length of the spinal canal (A-E) with the highest degree at the C6 level (C). Left lateral funiculus and dorsal funiculi show mild myelin pallor longitudinally (A-E) with the highest degree at the C6 level (C). Anterior white commissure connecting bilateral funiculi at the C6 level also show severe myelin pallor. See the scheme in (F). An arrow in Figure 2A indicates the demyelinating foot process extending downwardly from the massive lesion of the medulla oblongata. (F) Schematic presentation of degrees and distribution of myelin pallor (C1 to T1). Yellow and ochre indicate grey matter. (G-I) Degrees of demyelination in different funiculi at the level of T1. Double stain with KB stain and neurofilament immunoperoxidase. Severe demyelination in the right lateral funiculus (G) and mild demyelination in the right dorsal funiculus (H) in comparison to myelinated axons in the right anterior funiculus (I). J-L. Right lateral funiculus. (J) T lymphocytes are infiltrated into the parenchyma. CD3 immunoperoxidase. (K) CTLs are infiltrated into the parenchyma. CD8 immunoperoxidase. (L) Activated microglia in the right funiculus. CD68 immunoperoxidase. Thick scale bar = 2 mm. Thin scale bar = 40 μm.

At the lower end of the medulla oblongata the massive lesion also occupied the left ventral half and extended to the right. Multiple slender processes branched from the main part of the lesion extended downwardly (Figures 2G-2I), and the longest of which was to the C1 level (Figure 4A), which were clearly visible with KB stain and high-iron diamine stain (Figure 2I). Bilateral dorsal portions are relatively unaffected including cranial nerve nuclei and sensory nuclei, cuneate nucleus, and gracile nucleus. However, sensory tracts of cuneate fasciculus and gracile fasciculus were mildly demyelinated (Figures 2G, 2H). Left portion of pyramidal tract and pyramidal decussation was involved in the massive lesion, and the right pyramidal decussation showed mild demyelination (Figures 2G, 2H).

Spinal cord

**Figure 5:**
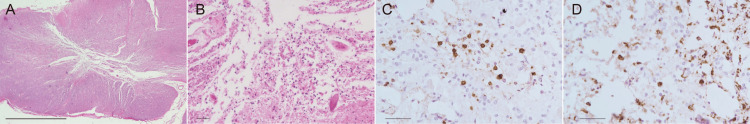
Histopathology of the spinal cord at the level of C6. (A) The spinal cord is deformed horizontally as a toothpaste artefact due to the collapsed gray matter. (B) The grey matter is collapsed with lymphocytic infiltration. (C) Many CTLs are infiltrated into the grey matter. CD8 immunoperoxidase. (D) Microglia increase in number and size in the collapsed grey matter. CD68 immunoperoxidase. Thick scale bar = 2 mm. Thin scale bar = 40 µm.

In the spinal cord, lateral funiculi and dorsal funiculi showed mild to moderate myelin pallor in their full length, C1 to S5 (Figures 4A-4F). Although the axons revealed with neurofilament peroxidase were preserved well, the intensity of myelin pallor was markedly different among the funiculi (Figures 4G-4I). The lesion of right lateral funiculus is connected to the massive lesion in the medulla oblongata. Right lateral funiculus demyelinated moderately, provided that the degree of demyelination of the medulla oblongata was severe. However, at the C1 level the degree was as mild as the pyramidal decussation, and the demyelination of right funiculus became moderate beneath C2 to S5 (Figures 4A-4E). Demyelination of dorsal funiculi and left lateral funiculus were mild (Figures 4A-4F). Anterior funiculi were not affected. By immunoperoxidase C3+/CD8+ CTLs were scattered in the parenchyma (Figures 4J, 4K) and the density of CTLs is almost coincident with the degree of demyelination. CD4 helper T-cell was not detected in the parenchyma. Numerous CD68+ ramified and ameboid microglia were found in the parenchyma (Figure 4L). The degree of demyelination of the funiculi was also different among the levels of the spinal cord. Demyelination at C5 to C7 levels is the severest in each funiculus (Figures 4A-4F). The gray matter was collapsed at the level of C5 to C7 with marked loss of glial matrix in anterior, lateral, and posterior horns bilaterally (Figures 5A, 5B) accompanying demyelination of anterior white commissure (Figures 4C, 4F), and connecting bilateral demyelinating lesions in lateral funiculi. The location was coincided with the level of severe spinal stenosis pointed out before by MRI, although not being considered the direct cause of the lesion at autopsy.

By H&E stain the entire white matter of the spinal cord showed no morphological change, although KB stain with neurofilament immunoperoxidase showed demyelination. CTLs (Figure 5C) and activated microglia (Figure 5D) were also in the collapsed parenchyma of the gray matter. Perivenular CTLs are found in the grey matter especially at C5 to C7 level. CD4+ helper T lymphocytes and CD20+ B cells were found around vessels in the gray matter but not found in the funicular parenchyma. Perivenular CTLs were also found in funiculi.

Cerebrum

**Figure 6:**
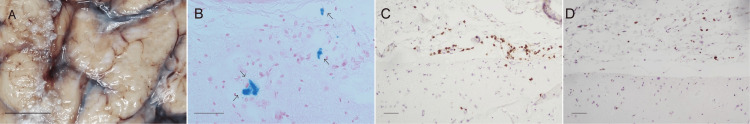
Leptomeningitis and subpial hemorrhage. (A) Hemosiderin-laden macrophages (arrows) in the leptomeninges (upper two). (B) The superficial cortex (lower two) with Berlin blue stain. (C) Band-like infiltration of CD8+ CTLs in the pia mater. CD 8 immunoperoxidase. D. Diffuse infiltration of CD68+ macrophages. CD68 immunoperoxidase. Thick bar = 2 mm. Thin scale bar = 40 µm.

Gross examination of the brain surface showed the clouded leptomeninges and hemorrhage along with subpial veins and arteries (Figure 6A). microscopically extravasation of red blood cells and hemosiderin-laden macrophages in the pia mater and the superficial cortex (Figure 6B). CTLs were located perivenular and in the pia mater (Figure 6C), and CD68+ macrophages were diffusely in the pia mater (Figure 6D).

**Figure 7:**
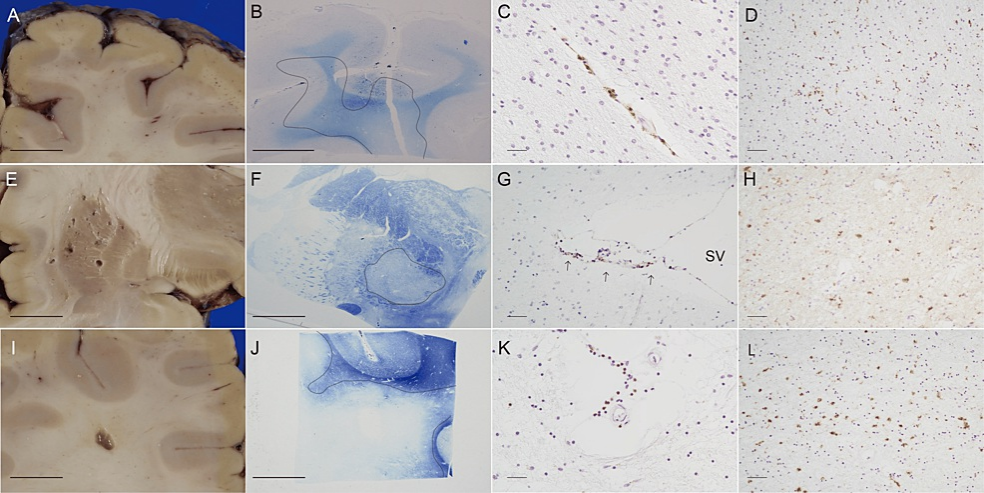
Perivenous demyelination in the cerebrum. A-D. Right occipital lobe. Dilated perivascular spaces (DPS) and ochraceous discoloration (A) showing demyelination by KB (B, dotted line). Perivenular cuff of CD8+ CTLs (C) and CTLs in the parenchyma in the white matter (D). E-H. Right lenticular nucleus. Right lenticulate nucleus showing the putamen with lacunar infarcts and DPS, and the globus pallidus and internal capsule without an appreciable change (E)€. A demyelinating faint zone by KB in and around the inner segment of globus pallidus (F, dotted line). Perivenular cuff of CD8+ CTLs (G, arrows, venule; SV, small vein). CD68+ ameboid and ramified microglia in the parenchyma (H). I-L. Right frontal lobe. A lacunar infarct and DPS in the white matter (I), and a large demyelinating area by KB stain (J) around the infarct. Perivenular cuff of CD8+ CTLs (K), and active microglia (L) in the demyelinating area. Thick scale bar = 1 cm. Thin scale bar = 40 µm.

Gross examination of slices of the cerebrum showed lacunar infarcts in the basal ganglia and deep white matter and numerous dilated perivascular spaces (DPS). Perivascular spaces with bleeding were scattered but they were fewer than those seen in MRI taken before death. Faint geographic discoloration with ochreous tints were found in the white mater with a carefully elaborated examination (Figure 7A), that was demyelination shown by KB stain (Figure 7B). It was associated with DPS in the white matter just beneath the cortex and extended over the deep cortex (Figure 7B). Microscopic DPS were found diffusely in the demyelinated lesion. They are mainly venules and cuffed with CD8+ CTLs (Figure 7G). DPS were also found in small veins and arterioles and small arteries. CD68+ macrophages were in dilated DPS and activated CD68+ microglia with ramified and ameboid features at the demyelinated parenchyma (Figure 7H). A few CD8+ CTLs were also scattered in the demyelinating parenchyma.

In the basal ganglia a few lacunar infarcts up to 5 mm in diameter with perivascular necrosis and macroscopically visible DPS 0.3 to 3 mm in diameter without perivascular necrosis were distinct especially in the putamen (Figure 7E). Lacunar infarct had a small artery and other types of small vessels in the center. They were surrounded by a few lymphocytes. Numerous microscopic DPS were associated with the macroscopic DPS (Figure 7E), and the vessels, mainly venules, were markedly cuffed with CTLs. Demyelination was also found in the white matter adjacent to basal ganglia and the gray matter (Figure 7E). Vessels of microscopic DPS were surrounded by lymphocytes, mainly CD8+ CTLs, and CD68+ macrophages. Perivascular cuffs with CTLs were marked in venule (Figure 7G). CD68+ ramified and ameboid microglia were also found in the parenchyma of the gray matter (Figure 7H).

A few lacunar infarcts were also found in the white matter, and the small infarct was in the center of the large demyelinating lesion (Figures 7I, 7J). There were residual structure of vessels including small artery in the center of the infarct. Although a few CTLs were around the small artery, CTL infiltration in the perivenular spaces at the demyelinated area was more distinct (Figure 7K). CD68+ ramified and ameboid microglia were also found in the parenchyma of the white matter Macroscopic and microscopic DPS are diffusely found in the cortex, white matter, and basal ganglia especially in the boundary area of the cortex and medulla, and basal ganglia.

The foci of fresh and organized bronchopneumonia were observed in the right lung. In a focus of fresh bronchopneumonia multinucleated giant cells were found, leading to the diagnosis of repeated aspiration pneumonia. Bilateral kidneys showed marked dilatation of pelvis with the shape of mold of staghorn calculus and microscopically chronic pyelonephritis. In the urinary bladder, 14 stones up to 1 cm in diameter were found. The cause of death was sepsis due to these lesions.

Lung and urinary tracts

Ancillary Test for Detection of SARS-CoV-2 Using Formalin-Fixed Tissues

Real time RT-PCR was performed at the Department of Neurology, Kanazawa University according to the standard protocol using N1/N2 primers. Formalin-fixed tissue with or without paraffin-embedding of the massive lesion in the medulla oblongata, olfactory bulb, rhinencephalon of the frontal lobe, and C5-C7 of the spinal cord were used for detection of SARS-CoV-2. The virus was not detected. Immunostaining for SARTSCoV2 was also performed using formalin-fixed and paraffin-embedded tissue sections and two different anti-SARS-CoV-2-antibodies, affinity-purified rabbit polyclonal antibodies (NB100-56576, Novus Biologicals, CO, USA) and mouse monoclonal antibody (GTX632269, GeneTex, CA, USA), and the results were negative.

## Discussion

Numerous perivenular DPSs adjacent to macroscopic DPSs and perivenular cuff with CTLs were characteristic features in the cerebrum and pons. Furthermore, the onset course of punctate lesions with or without bleeding at different times and places suggested acute venular lesions caused by SARS-CoV-2 superimposed on chronic small vessel disease. On the other hand, the visible macroscopic findings in the cerebrum were lacunar infarcts and the macroscopic DPSs in the basal ganglia and deep white matter, which are common in the elderly with hypertension or diabetes mellitus and small vessel disease of small arteries and arterioles are suggested. The present case had hypertension and diabetes mellitus and old lacunar infarcts were shown by MRI at the early time of COVID-19 pneumonia. The distribution pattern of DPS in basal ganglia and deep white matter at the autopsy implied the hypoxic change due to reduced arterial flow, and similar cases with COVID-19-related encephalopathy have been reported. The coincident distribution of arterial disease and venular disease should be the consequence of the increased expression of ACE2 in the ischemic area as shown in an animal ischemia model [5].

Microvascular injury in COVID-19 has been well-documented [5]. It was established that the membranous receptor of ACE2 in endothelial cells binds to SARS-CoV-2 [12]. Moreover, a mouse model clarified that the spike protein of pseudovirion without RNA caused endothelial cell damage [13]. The microvascular injury could be caused by endothelial cell damage through immune-induced or pauci-immune mechanisms [12]. Another root could be pericytes [13] of postcapillary venules or smooth muscle cells of larger venules. Pericytes, which play a crucial role in maintaining vascular integrity, express a high level of ACE2 in the brain [14] and are a possible target of SARS-CoV-2.

Medulla oblongata is the favorite location of SARS-CoV-2, at which the virus is frequently detected [15]. The virus was not detected in the CNS of the present case at autopsy probably due to the long interval between the symptom onset and death. Despite the negativity at autopsy, the possible entry routes of SARS-CoV-2 at the onset of neurological symptoms are hematogenous and neurogenous. There is clear evidence that SARS-CoV-2 enters the brain through the olfactory nerve and olfactory bulb followed by transsynaptic spreading to the medulla oblongata [15]. In our case, since mild demyelination and CTL reaction in the parenchyma of the olfactory bulb without vascular change was found, it is highly probable that the virus penetrated through a similar route. The hematogenous route from the lung or other infected tissues might be also involved in establishing the peculiar lesion cooperated with the effect through the neurogenous route. Another possible route is that from the fourth ventricle, but it is unlikely because the ventral area of the medulla oblongata was unaffected.

So-called demyelinating pseudotumor in the sense of non-neoplastic lesion may be applied for the lesion of the medulla oblongata but includes distinct disease entities including tumefactive MS [16]. The massive demyelinating lesion was characterized by mixed perivenous and confluent demyelination, glycosaminoglycan-rich matrix with a decrease in astrocytic endfeet and altered neuronal cells. Both colloidal iron, which is a sensitive stain for hyaluronic acid and sulfated glycosaminoglycans, and high-iron diamine, which visualizes sulfated glycosaminoglycan, were positive. Matrix-type glycosaminoglycans in the CNS are hyaluronic acid and chondroitin sulfate [17], and then the increased matrix components are considered chondroitin sulfate and hyaluronic acid, which are recently known to be components of perineural nets. Hyaluronic acid is synthesized by glial cells [18], and chondroitin sulfate proteoglycan is by both neuronal [18] and glial cells [19]. It is quite mysterious that the massive lesion was filled with the perineural nets that originally existed as a pericellular thin layer. The nets could be produced by neuronal cells, presumably vacuolating round cells, in the process of establishment of the lesion. So many vacuolating round cells were alive that the perineural nets produced might act as a supportive and protective scaffold [17].

ADEM is an inflammatory demyelinating disorder, and the pathogenesis of ADEM has been presumed autoimmune [9]. The microscopic characteristic feature of sleeves of demyelination around venules associated with inflammatory infiltration in the present cerebrum was consistent with that of ADEM, but manifestation in the medulla oblongata and the spinal cord was not of ADEM.

Transverse demyelination over the entire spinal cord was like longitudinally extensive transverse myelitis reported in COVID-19 [2] We further clarified the longitudinal demyelination in the right pyramidal tract was connected to the lesion of the right medulla oblongata through pyramidal decussation, which means it is a secondary change to the massive lesion of the medulla oblongata. The longitudinal lesion along with a long axon through the medulla oblongata to each level of the spinal cord suggested axonal transport. It is well-known that neuronal cells do not express MHC I on their surfaces, and possibly escape from a direct attack by CTL, which enables free transfer in a neuronal cell. However, MHC I is expressed in immune activation such as viral infection [8]. The viral antigens presented by MHC I are recognized by T cell receptors on CTL, leading to direct cell injury. Once the axon is injured, the viruses will be released into the environment, which in turn infect astrocytes and oligodendrocytes [20]. Glia also highly expresses MHC I, probably to a higher degree than neuronal cells. Thus, the lesion of demyelination with partial loss of neuronal axon pursues. Furthermore, demyelination of left funiculi and bilateral dorsal funiculi could be explained by the lesion of grey matter and anterior white commissure connected right and left in C5 to C7, which are presumed to be caused by augmented inflammation by preexisting injury by spinal canal stenosis. The preexisting inflammation augments the expression of MHC I as well as ACE2.

## Conclusions

The present case had different three types of demyelination, possibly reflecting differences in the route of viral transmission. The first type is perivenous demyelination in the brain and pons, as is shown in ADEM. The second one is unusual perineural nets-rich demyelination, which has not been reported histologically, in the so-called demyelinating pseudotumor of the medulla oblongata. The third one is confluent demyelination or longitudinal extensive demyelination in the spinal canal, as is shown in neuromyelitis optica spectrum disease and MS.
